# Evaluating the impact of covariate lookback times on performance of patient-level prediction models

**DOI:** 10.1186/s12874-021-01370-2

**Published:** 2021-08-28

**Authors:** Jill Hardin, Jenna M. Reps

**Affiliations:** 1 Observational Health Data Sciences and Informatics Community, New York, NY USA; 2 Department of Epidemiology, Janssen Research & Development, LLC, 1125 Trenton-Harbourton Road, Titusville, NJ 08560 USA

**Keywords:** Patient-level prediction, Feature extraction lookback periods

## Abstract

**Background:**

The goal of our study is to examine the impact of the lookback length when engineering features to use in developing predictive models using observational healthcare data. Using a longer lookback for feature engineering gives more insight about patients but increases the issue of left-censoring.

**Methods:**

We used five US observational databases to develop patient-level prediction models. A target cohort of subjects with hypertensive drug exposures and outcome cohorts of subjects with acute (stroke and gastrointestinal bleeding) and chronic outcomes (diabetes and chronic kidney disease) were developed. Candidate predictors that exist on or prior to the target index date were derived within the following lookback periods: 14, 30, 90, 180, 365, 730, and all days prior to index were evaluated. We predicted the risk of outcomes occurring 1 day until 365 days after index. Ten lasso logistic models for each lookback period were generated to create a distribution of area under the curve (AUC) metrics to evaluate the discriminative performance of the models. Calibration intercept and slope were also calculated. Impact on external validation performance was investigated across five databases.

**Results:**

The maximum differences in AUCs for the models developed using different lookback periods within a database was < 0.04 for diabetes (in MDCR AUC of 0.593 with 14-day lookback vs. AUC of 0.631 with all-time lookback) and 0.012 for renal impairment (in MDCR AUC of 0.675 with 30-day lookback vs. AUC of 0.687 with 365-day lookback ). For the acute outcomes, the max difference in AUC across lookbacks within a database was 0.015 (in MDCD AUC of 0.767 with 14-day lookback vs. AUC 0.782 with 365-day lookback) for stroke and < 0.03 for gastrointestinal bleeding (in CCAE AUC of 0.631 with 14-day lookback vs. AUC of 0.660 with 730-day lookback).

**Conclusions:**

In general the choice of covariate lookback had only a small impact on discrimination and calibration, with a short lookback (< 180 days) occasionally decreasing discrimination. Based on the results, if training a logistic regression model for prediction then using covariates with a 365 day lookback appear to be a good tradeoff between performance and interpretation.

**Supplementary Information:**

The online version contains supplementary material available at 10.1186/s12874-021-01370-2.

## Background

Observational healthcare data consists of timestamped data, which needs to be converted into features for a prediction model. Due to the temporality of the observational data, it is possible to either fully preserve the temporal nature of the data (‘temporal features’, for example as a feature matrix per patient with rows corresponding to medical events and columns corresponding to time and the entries being the medical event value at the specific time) or create a summary of the patient’s history (‘non-temporal features’, a feature vector per patient corresponding to medical events and the entries are the values, for example binary values indicating the presence or absence of an event in the patient’s history). Temporal features can be used with classifiers such as neural networks (deep learning) however, this is not possible with many conventional classifiers (such as logistic regression). In addition, there are difficulties when developing models using temporal data from healthcare claims and electronic healthcare record databases as the data come from a diversity of sources and are recorded at irregular frequencies with data often sparsely represented. This can present issues to classifiers such as neural networks when implementing the feature engineering [1], especially if the data are not large. In this paper we therefore focus on engineering non-temporal features.

**Fig. 1:**
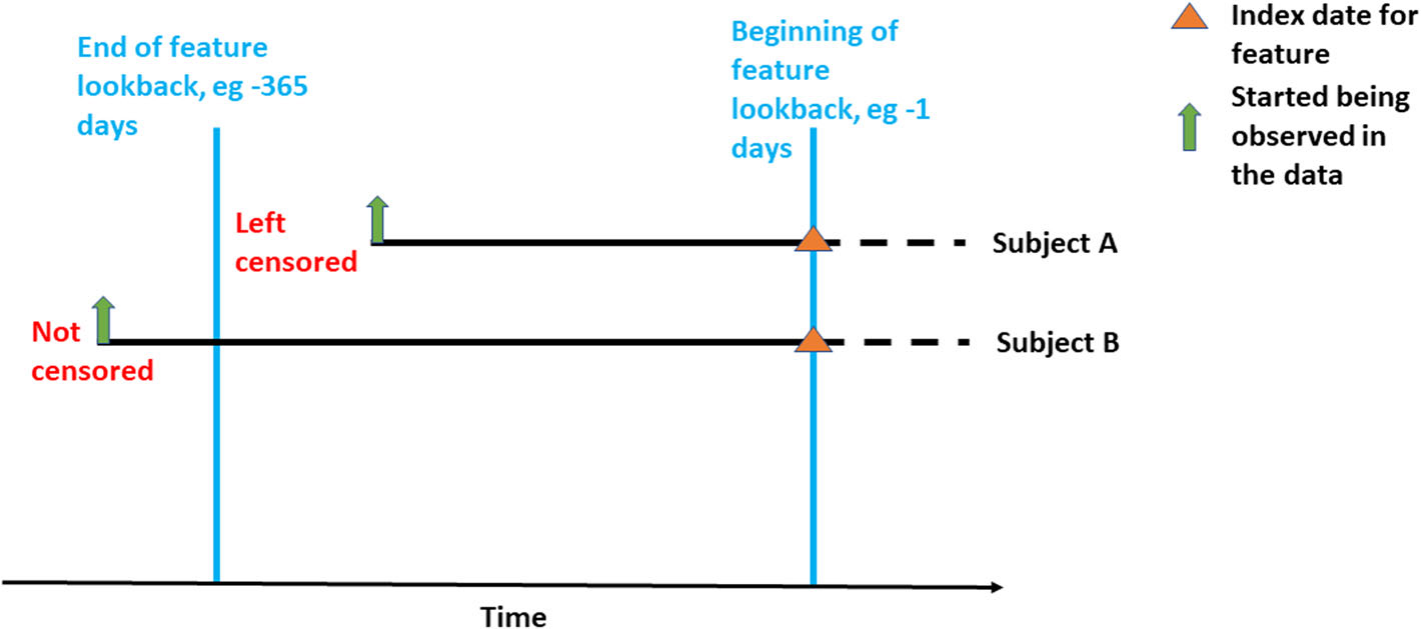
Left censoring and feature construction

Converting observational data to non-temporal data requires specifying a static lookback time where the value of the medical event is observed during the lookback period. It is possible to specify the lookback time, such as 365 days prior to index which means only the data recorded in the 365-days prior to index per patient are used when constructing the features. Alternatively, the lookback window can be specified to include all time prior, meaning all data recorded prior to index are used to construct the features. The benefits of using a longer look back are that you have a more complete picture of each patient, but there are multiple negative aspects including: (i) you treat a recent illness the same as an illness experienced years ago, (ii) you may have issues with left censoring as patients often do not have the same length of complete lookback (iii) you may run into issues when implementing the model in a new healthcare system if the mean complete lookback is shorter and (iv) if using all lookback time realistically patients have varying lengths of lookback which hinders interpretation. Figure 1 represents a subject with left censoring (subject A) and a subject without left censoring (subject B). For subject B there is no missing data in the feature construction, but for subject A the left censoring means we are unable to observe her for part of the lookback time (effectively missing data).

Studies using administrative data and investigating variations in the length of lookback period have been conducted in the context of incidence and effect estimation [2–4]. In a study of cancer cumulative incidence estimation the authors recommended using lookback of 2 or more years and discouraged the use of 1 year lookback but caveated that it is not possible to provide general recommendations as lookback period is dependent on the characteristics of the cancer site and the available data and the underlying research question [3]. A Korean study using a cohort database and examining lookback and estimating incidence of three gynecological diseases (uterine leiomyoma, endometriosis, and adenomyosis) found that as the lookback increased the proportion of misclassified incident cases decreased but advised that the optimal lookback for annual incidence depended on the nature and the stage of the respective diseases [4]. A comparative effect study using the Medicare beneficiary database and evaluating the effect of statin initiation on incidence of cancer recommended that a 3 year lookback was best but if infeasible that all available lookback is preferable to short fixed lookbacks [2]. Although these studies do not utilize the PLP methodology they illustrated that longer lookback reduces data noise for the diseases examined.

Few studies have evaluated the impact of the selection of the length of lookback time in the setting of predictive ability [5–7]. In a Korean study with data from the National Health Insurance Database evaluating in hospital mortality for patients aged 40 and older who underwent percutaneous coronary intervention the authors’ compared comorbidity measurements (Charlson comorbidity index, Elixhauser’s comorbidity, and comorbidity selection) using 3 years of inpatient records compared to models using 1 year of inpatient records and concluded the longer lookback period offered no improvement in predictive capacity [6]. Evaluation of the impact of 1 year vs. 2 year lookback in Charlson score for mortality among elderly Medicare beneficiaries using claims data reported nearly identical C-statistics [7]. An Australian study using population based hospital data examined prediction of hemorrhage in pregnancy among eight different chronic disease cohorts and evaluated six lookback periods and concluded that although longer ascertainment periods resulted in improvement of identification of chronic disease history it did not change the resulting C-statistics [5]. These studies evaluated a limited set of outcomes (mortality and hemorrhage during pregnancy). Based on the findings of these studies for the outcomes evaluated lookback period did not materially impact the results.

The intent of this study is to evaluate the impact of several lookback periods on the performance of models predicting two acute and two chronic diseases. Multiple databases are included in the study to investigate both internal and external performance. This research may help identify recommendations for the optimal lookback period for these outcomes using administrative data. We hypothesize that using a 365 days prior lookback will result in well performing prediction models that are more transportable and interpretable across databases as this is a trade-off between gaining a sufficient picture of each patient’s health history while reducing issues with left censoring.

## Methods

We used the OHDSI PatientLevelPrediction framework [8] and R package to develop and evaluate the prediction models in this study (Table 1).

**Table 1:** Counts of population, percentage with outcome by database

Outcome	Database	Total Population (N)	Outcome Percentage (%)
Stroke	CCAE	688,011	0.9
Stroke	OPTUM	555,849	2.3
Stroke	MDCD	166,774	2.9
Stroke	MDCR	46,696	4
Stroke	Panther	1,667,849	1.8
Gastrointestinal bleeding	CCAE	700,384	1.6
Gastrointestinal bleeding	OPTUM	556,913	2.5
Gastrointestinal bleeding	MDCD	178,419	3.6
Gastrointestinal bleeding	MDCR	51,084	2.8
Gastrointestinal bleeding	Panther	1,688,161	1.7
Renal impairment	CCAE	721,671	1.8
Renal impairment	OPTUM	536,981	4.2
Renal impairment	MDCD	164,979	5.3
Renal impairment	MDCR	49,495	5.2
Renal impairment	Panther	1,608,521	4.2
Diabetes	CCAE	587,205	3.1
Diabetes	OPTUM	458,033	3.8
Diabetes	MDCD	128,517	4.6
Diabetes	MDCR	42,306	3.1
Diabetes	Panther	1,294,517	2.6

### Data

We developed models using five US observational datasets. Each dataset has unique attributes. The IBM MarketScan® Commercial Claims and Encounters Database (CCAE) which contains insurance claims for commercially employed individuals and their dependents and contains subjects less than or equal to the age of 65. The Optum® De-Identified Clinformatics® Data Mart Database – Socio-Economic Status – (Optum) is a similar database to CCAE except that it also contains claims from subjects with Medicare supplemental insurance and thus does not have an upper age threshold. IBM MarketScan® Medicare Supplemental and Coordination of Benefits Database (MDCR) database contains claims from subjects with Medicare supplemental insurance and thus contains subjects 65 years and older. The IBM MarketScan® Multi-State Medicaid Database (MDCD) contains claims from subjects covered by Medicaid and is primarily composed of women and children. The Optum® de-identified Electronic Health Record Dataset (Panther) dataset is an electronic health records (EHR) and contains information derived from clinical Notes using Natural Language Processing (NLP). All databases were transformed to the Observational Medical Outcomes Partnership Common Data Model version 5.3.1. The use of IBM and Optum databases were reviewed by the New England Institutional Review Board (IRB) and were determined to be exempt from broad IRB approval.

### Study population

We extracted data for patients who are newly treated with a hypertensive medication to predict four outcomes occurring from 1 day to 365 days after their first prescribed hypertensive treatment.

The target population was new users of hypertensive medications and the eligibility was defined as first time exposure to one or more hypertensive medications on or after 2013 with at least one diagnosis of hypertensive disorder in the 365 days prior to the index drug exposure. We excluded subjects with a prior diagnosis of any of the outcomes evaluated. We required subjects to have at least 365 days of continuous observation prior to the index date. See Additional file 1 for the codes and logic used to define the target population. The 365 days minimum prior observation is a standard criterium when analyzing observational data, as this ensures there is data for each patient [9]. In addition, as the target population was newly treated patients, the minimum prior observation reduces the chance of a patient with a history of hypertensive treatment being incorrectly included when they first are observed in the database even though they have a long history of hypertensive treatment (as this will not be observed in the data). The impact of the minimum prior observation is investigated via a sensitivity analysis (we investigated 0 days and 730 days prior observation requirements) see Additional file 2.

Two of the outcomes were acute health conditions (stroke and gastrointestinal bleeding) and two were chronic health conditions (diabetes and renal impairment) from 1 day after index until 365 days after index. Eligibility for the outcome populations included the first occurrence of gastrointestinal bleeding or stroke or diabetes or renal impairment. See Additional file 1 for the codes and logic used to define the outcome populations.

### Candidate predictors

Candidate non-temporal features were engineered from the administrative claims data that exist on or prior to the target index date and followed a standardized feature construction process [8]. These variables were demographics, visit type, binary indicators of medical events and counts of record types. The demographics included gender, race and ethnicity (where available), age in 5-year groups (0–4, 5–9, 10–14,…, 95+) and month at the target index date. Binary indicator variables were created for medical events based on the presence or absence of each within the clinical domains of conditions, drugs, and procedures within several time periods: 14, 30, 180, 365, 730 days, and all time prior to index. As binary covariates are the presence or absence of records for various conditions or drugs during time intervals, missing values will not be contained in the covariates. This is because when a patient does not have a condition recorded, we cannot distinguish whether it is due to the patient having the condition but not having it recorded (missing) or them not experiencing the condition. Therefore, missing records for condition, drugs or procedures are treated as the patient not having the condition, drug, or procedure. Left censoring may result in missing records. Age and gender are mandatory in the OMOP common data model (CDM) [10], so are never missing. If a database contains race/ethnicity it will be recorded for all patients.

The published best practices for model development were followed [8]. To enable full transparency and implementation by other researchers using different data all the definitions, analysis code, and prediction models are available in the OHDSI github repository: https://github.com/ohdsi-studies/PredictionCovariateLookback.

### Development and validation of prediction model

**Fig. 2:**
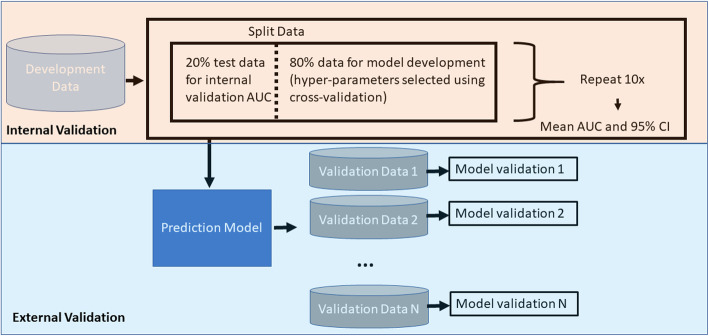
Model development and internal and external validation process

In this study we focused on developing logistic regression with LASSO regularization (LASSO logistic regression) binary classifiers [11]. LASSO logistic regression is a good classifier to use when there is a large number of covariates. We used a test/train split with cross validation design to develop each model. Data for the whole population were split into a 20 % test dataset and 80 % training dataset. The training dataset was used to learn the model (the optimal regularization hyper-parameter was selected using 10-fold cross validation using the train data) (Fig. 2). The test dataset was used to internally validate the model.

As the test/train split can impact performance, we repeated the process by developing 10 models per < lookback period, database, outcome > combination using different test/train splits. In total we developed 1400 models as we investigated 7 lookback periods (14 days, 30 day, 90 days, 180 days, 365 days, 730 days, and all days prior to index), 5 databases and 4 outcomes and repeated the test/train split 10 times. This provided a distribution of performance estimates for each < lookback period, database, outcome > combination.

To evaluate each model’s performance we calculated discrimination using the area under the receiver operating characteristic curve (AUC) metric and calibration using the calibration intercept and slope. As model development was repeated 10 times (using different test/train splits) a mean AUC and confidence interval were calculated for internal performance estimates.

To investigate the impact of the lookback period on external validation performance, the 10 models per < lookback period, database, outcome > were inspected and the model with the highest internal discrimination was externally validated across the other four databases.

Model complexity was also investigated in addition to performance, based on the number of predictors selected into the model. This enabled the investigation of whether the lookback has any impact on model complexity.

## Results

This study examined model performance over seven lookback periods (14, 30, 90, 180, 365, 730, and all time prior to index) for two chronic (diabetes and renal impairment) and two acute (stroke and gastrointestinal bleeding) outcomes in subjects newly treated with hypertensive medications across five US databases.

**Fig. 3:**
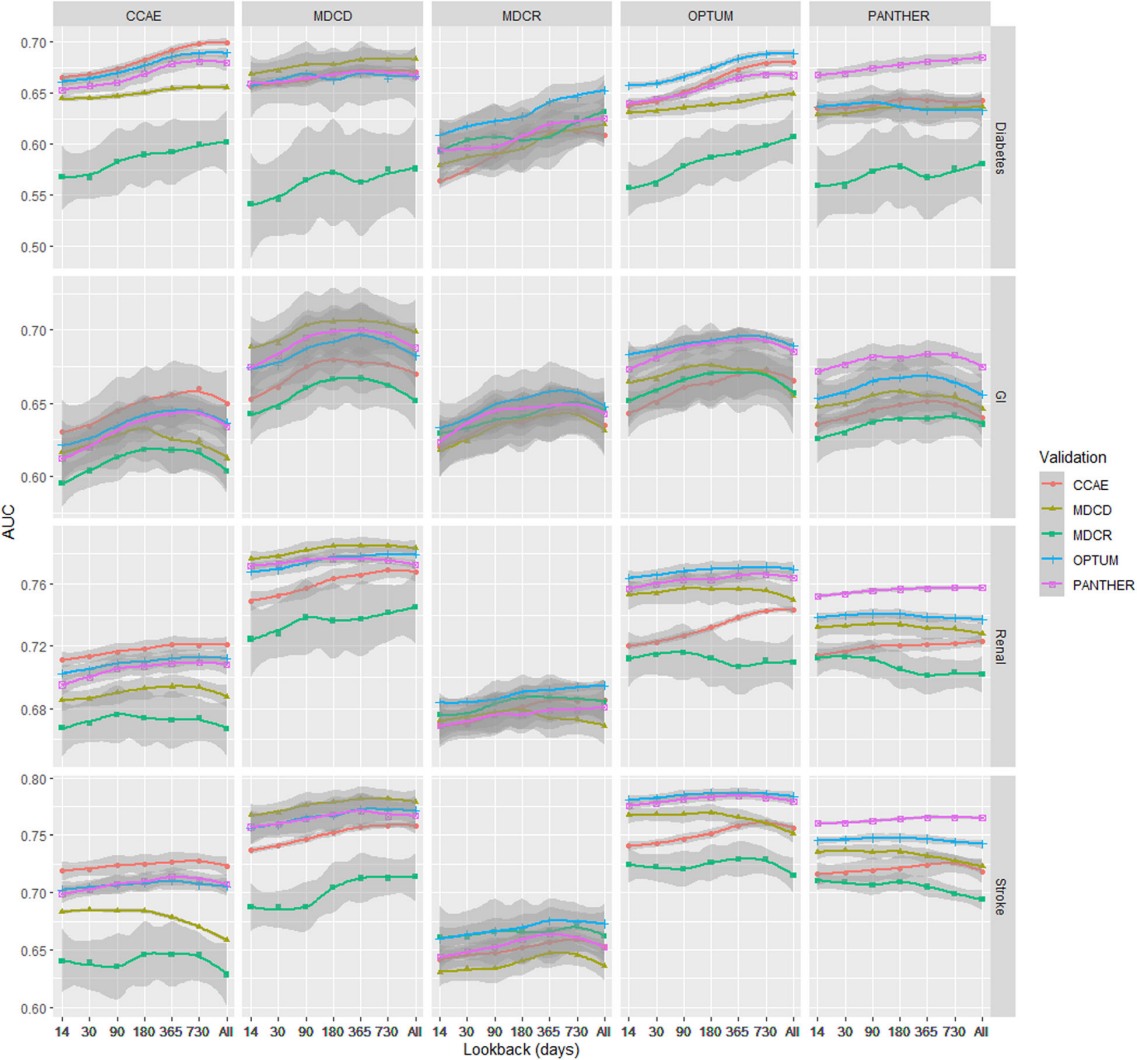
Mean AUC across two chronic (Diabetes and Renal Impairment) and two acute (Gastrointestinal bleeding and Stroke) cohorts over five US databases. The database at the top was used to train the model. Colors and shapes indicate the database used to compute the AUC. The shaded area indicates the range of AUCs observed across the 10 replications

Figure 3 shows the internal and external validation discrimination (mean AUC) across the databases for all four models. The rows correspond to the different outcomes and the columns correspond to the database used to develop the model. There was generally a positive correlation between covariate lookback and AUC, but differences were observed across outcomes. Additional file 6: Fig. 5 shows similar trends for the discrimination metric area under the precision recall curve (AUPRC) but the values are lower as the precision is impacted by the outcome rareness.

The two chronic illnesses, diabetes (top row) and renal impairment (third row), show an increasing AUC over the lookback periods up to 365 days (14, 30, 90, 180 days) and then remained stable or improved marginally in CCAE, MDCD, Optum, Panther, and MDCR (renal impairment only). The mean AUC for diabetes models results from MDCR illustrated a continuous increase. The external AUCs for renal insufficiency occasionally decreased in performance when an all-time prior lookback is used. Although the AUC differed across lookbacks, the max difference in AUC across lookbacks within a database was < 0.04 (0.593 with 14-day lookback in MDCR vs. 0.631 with all-time lookback in MDCR) for diabetes and 0.012 for renal impairment (0.675 with 30-day lookback in MDCR vs. 0.687 with 365-day lookback in MDCR).

For the acute illnesses, the mean AUC for stroke (fourth row) was relatively stable across the lookbacks and for gastrointestinal bleeding (second row) it increased slightly up until a 365/730 day lookback and then decreased. The max difference in AUC across lookbacks within a database was 0.015 (0.767 with 14-day lookback in MDCD vs. 0.782 with 365-day lookback in MDCD) for stroke and < 0.03 for gastrointestinal bleeding (0.631 with 14-day lookback in CCAE vs. 0.660 with 730-day lookback in CCAE). Additional file 3 shows all numeric results for the internal and external validation discrimination across all cohorts and databases. Additional file 4 includes additional metrics specifically intercept and slope metrics by lookback days for internal and external validated models.

The mean number of predictors in internally validated models by lookback days in the chronic outcome cohorts (diabetes and renal impairment) increase with lookback time and the largest number of predictors were observed in CCAE and the smallest number of predictors were found in MDCR (Additional file 5: Fig. 4). The acute outcome cohorts (stroke and gastrointestinal bleeding) illustrate the same pattern of mean number of predictors as the chronic outcome cohort models however overall fewer numbers of predictors are included in the final models.

The sensitivity analyses varying the prior lookback days did not result in changes in internally validated model performance. Full results for the sensitivity analyses can be accessed in Additional file 2.

## Discussion

The objective of this study was to provide empirical evidence into the impact that the choice of non-temporal feature lookback windows has on model performance by investigating two acute and two chronic disease outcomes across five databases.

The main findings of our results are that the impact of lookback is highly dependent on the healthcare outcome investigated. In the outcomes investigated in this study the differences in AUC values across lookbacks were generally small for renal insufficiency and stroke, indicating the choice of lookback makes little impact in discrimination for some outcomes. A lookback of 365 days was a reasonable trade-off between maximizing discriminative ability, minimizing the model complexity, and increasing interpretation. Specifically, interpretation is nebulous when using all time prior lookback because it can vary by patient in a database (i.e., a patient may be observed for 2 years or 20 years) so the results are more interpretable when using a shorter lookback, such as 365 days or 180 days where many subjects will have at least this length of lookback. Use of a distinct well defined time period, e.g. 365 days may improve model transportability because databases have different observation periods (some databases may have 1–5 years of observation per patient, others may have 10–50 years observation). The real ‘all time prior’ can therefore differ across databases, but 365-day lookback is likely found in most databases. If model development time is restricted then using a 365 day lookback is reasonable because it makes little impact on performance (for these outcomes and model methodology), but if time is unrestricted and small gains in discrimination are valuable, then multiple lookbacks (or more advanced lookbacks) should be evaluated. This study found that lookback can make an impact if it is too short, but in general the lookback didn’t make a big impact on discrimination or calibration. Models developed using covariates with a 1-year lookback seemed to perform similarly to the model with the maximum lookback therefore we recommend using the 365 day lookback setting. In future work multiple or adaptive lookbacks, i.e. lookbacks varying by the type of covariate (condition vs. drug exposure) could be investigated.

### Overview of previous work

Studies of lookback periods have been conducted in the context of incidence estimation/phenotype development, effect estimation, with a limited number of studies in the context of prediction [2–7]. Incidence estimation and effect estimation studies all advise that longer lookback reduces data noise for the diseases and data sources evaluated [2–4]. The findings from the relatively few prediction studies stated that for the outcomes evaluated that lookback period did not materially impact the model discrimination but did improve identification of the disease history [5–7].

### Strengths and limitations

This study examined the impact of lookback period for two acute and two chronic outcomes on prediction model discrimination using administrative claims data. The data sources used afforded large sample sizes and long observation periods resulting in good precision and the ability to evaluate several lengths of lookback period.

There are different ways to address left-censoring including: (1) reduce the lookback period and include all patients, (2) reduce the lookback for just the left-censored, (3) remove patients with insufficient lookback or (4) impute the missing data for left-censored patients. In this study we effectively focus on a combination of first two approaches as the other approaches can cause generalizability issues or are computationally expensive [12].

Our study developed 1400 models focusing on two examples of chronic and acute outcomes, utilizes US administrative claims data, and uses a single prediction algorithm, the LASSO logistic regression. There is no guarantee that the trends observed in this study would generalize across all outcomes, models and data. Therefore, future research should focus on evaluation of additional outcomes, utilize alternative types of data sources, and evaluate additional prediction algorithms. The reason for the difference in performance between models with different lookbacks was not investigated and is an interesting area of future work. In this study, models were developed using a single fixed lookback (e.g., all condition/drugs shared the same lookback for a specific lookback setting). This choice was made to clearly measure the impact of length of lookback and due to only using one time period being a commonly reported modelling approach [13, 14]. However, combining multiple lookbacks or making the lookback adaptive to the condition/drug may lead to improved model performance and our results can be used as a benchmark for more advanced lookback heuristic methods.

Our study predicts new onset of illness and utilizes 365 days prior observation time to apply a washout window to confirm the absence of the illness and therefore it is possible that our study could suffer from informative presence [15] and could include data from sicker patients. Therefore, we include results from two sensitivity analyses where the minimum required previous observation was set to 0 days and set to 730 days in order to assess the impact of informative presence.

The algorithms used to identify the four outcomes likely are prone to some misclassification, although we performed no formal evaluation of the operating characteristics. We did inspect cohort definitions and characteristics using the CohortDiagnostics R package [16] prior to execution of the PLP models. Our study evaluated seven lookback periods but none in combination and thus it is possible that combinations of lookback periods could result in model discrimination improvement. Model performance utilizing different lookbacks may not be generalizable to alternative target and outcome populations, data sources, and prediction algorithms.

## Conclusions

The impact of lookback period was evaluated and the results of our study suggest for the two chronic outcomes evaluated that a lookback of at least 365 days be evaluated and that for the acute outcomes evaluated that a lookback of < = 365 days was sufficient to optimize model discrimination. We found lookback can have an impact on model discrimination if it is too short, but in general lookback did not impact discrimination or calibration. Logistic regression models developed using covariates with a 1-year lookback performed similarly to models with the maximum lookback and therefore we recommend a well defined lookback period (365 days) as opposed to all time lookback because all time varies among patients in observational databases as people can have variations in prior observation time. In future work multiple or adaptive lookbacks could be investigated.

## Supplementary Information

**Additional file 1.**

**Additional file 2.**

**Additional file 3.**

**Additional file 4.**

**Additional file 5.**

**Additional file 6.**

## Data Availability

The data that support the findings of this study are available from IBM MarketScan® and Optum® but restrictions apply to the availability of these data, which were used under license for the current study, and so are not publicly available. Data are however available from the authors upon reasonable request and with permission of IBM MarketScan® and Optum®. To enable full transparency and implementation by other researchers using different data all the definitions, analysis code, and prediction models are available in the OHDSI github repository: https://github.com/ohdsi-studies/PredictionCovariateLookback.

## Abbreviations

OHDSI: Observational Health Data Science and Informatics
PLP: Patient-level prediction
OMOP: Observational Medical Outcomes Partnership
CDM: Common data model
CCAE: Commercial Claims and Encounters Database
MDCR: Medicare Supplemental and Coordination of Benefits Database
MDCD: Multi-State Medicaid Database
EHR: Electronic health records
NLP: Natural Language Processing
IRB: Institutional Review Board
AUC: Area under the curve
