# Supplementary material for: Evaluating the impact of covariate lookback times on performance of patient-level prediction models
Source: BMC Med Res Methodol. 2021 Aug 28;21:180. doi: 10.1186/s12874-021-01370-2 (PMC8403343; doi:10.1186/s12874-021-01370-2)
Supplement: Supplementary file 2 — Additional file 2. [file 12874_2021_1370_MOESM2_ESM.docx]

**Appendix B, Figure 1. AUC sensitivity analysis requiring 0 days prior observation**


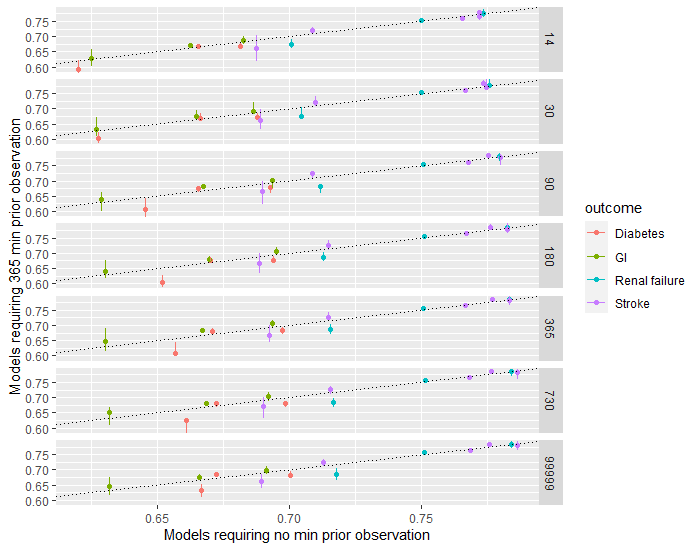
Figure 1 - the AUC value of the models that were trained using a population of paients that were required to have 0 days prior observation (x-axis) plotted against the AUC value of the models that were trained using a population of paients that were required to have 365 days prior observation (y-axis) matched on covariate lookback, outcome and database. The vertical lines are the confidence intervals of the AUC.

The x-axis is the model performance requiring 0 days prior observation and the y-axis is the model performance requiring 365 days observation and each dot is a paired outcome and database. The dots are distributed around the diagonal indicating that there is not a sufficient change in model performance. The results are restricted to internally validated model performances. (NOTE: 99999 indicates all time)

**Appendix B, Figure 2. Sensitivity analysis requiring 730 days prior observation**
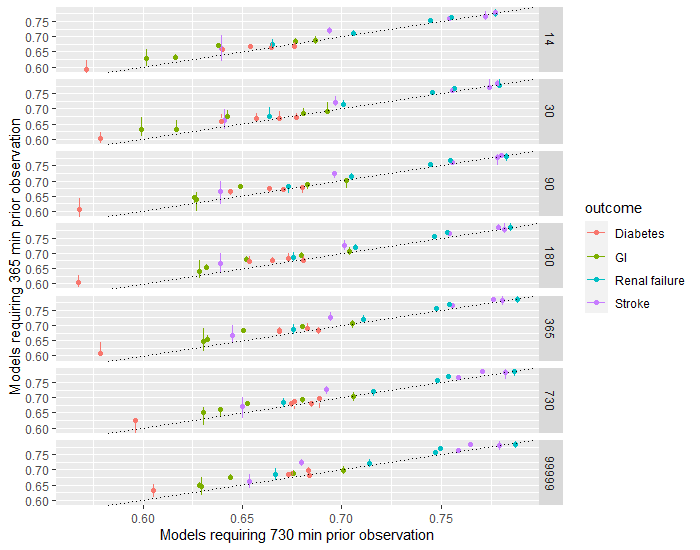
Figure 2 - the AUC value of the models that were trained using a population of paients that were required to have 730 days prior observation (x-axis) plotted against the AUC value of the models that were trained using a population of paients that were required to have 365 days prior observation (y-axis) matched on covariate lookback, outcome and database. The vertical lines are the confidence intervals of the AUC.

The x-axis is the model performance requiring 730 days prior observation and the y-axis is the model performance requiring 365 days observation and each dot is a paired outcome and database. The dots are distributed around the diagonal indicating that there is not a sufficient change in model performance. The results are restricted to internally validated model performances. (NOTE: 99999 indicates all time)

**Table 2. Percentage of subjects excluded due to minimum prior observation period requirement**

| **Outcome** | **Database** | **Total Population (N)** | **% Population with at least 365 days prior observation**  **(%)** | **% Population with at least 730 days prior observation**  **(%)** |
| --- | --- | --- | --- | --- |
| Stroke | CCAE | 688011 | 0.6943 | 0.5006 |
| Stroke | OPTUM | 555849 | 0.7513 | 0.5796 |
| Stroke | MDCD | 166774 | 0.6823 | 0.5243 |
| Stroke | MDCR | 46696 | 0.7971 | 0.6368 |
| Stroke | Panther | 1667849 | 0.7495 | 0.6772 |
| Gastrointestinal bleeding | CCAE | 700384 | 0.6705 | 0.4683 |
| Gastrointestinal bleeding | OPTUM | 556913 | 0.688 | 0.4915 |
| Gastrointestinal bleeding | MDCD | 178419 | 0.6663 | 0.4888 |
| Gastrointestinal bleeding | MDCR | 51084 | 0.7681 | 0.5927 |
| Gastrointestinal bleeding | Panther | 1688161 | 0.7912 | 0.7143 |
| Renal impairment | CCAE | 721671 | 0.5695 | 0.4068 |
| Renal impairment | OPTUM | 536981 | 0.5544 | 0.4037 |
| Renal impairment | MDCD | 164979 | 0.5229 | 0.3686 |
| Renal impairment | MDCR | 49495 | 0.635 | 0.4868 |
| Renal impairment | Panther | 1608521 | 0.7165 | 0.6318 |
| Diabetes | CCAE | 587205 | 0.3453 | 0.2207 |
| Diabetes | OPTUM | 458033 | 0.3126 | 0.2034 |
| Diabetes | MDCD | 128517 | 0.3061 | 0.19 |
| Diabetes | MDCR | 42306 | 0.3031 | 0.1979 |
| Diabetes | Panther | 1294517 | 0.5686 | 0.474 |
