# Supplementary material for: Evaluating the impact of covariate lookback times on performance of patient-level prediction models
Source: BMC Med Res Methodol. 2021 Aug 28;21:180. doi: 10.1186/s12874-021-01370-2 (PMC8403343; doi:10.1186/s12874-021-01370-2)
Supplement: Supplementary file 4 — Additional file 4. [file 12874_2021_1370_MOESM4_ESM.docx]

Calibration (intercept) plot for internal and external validation


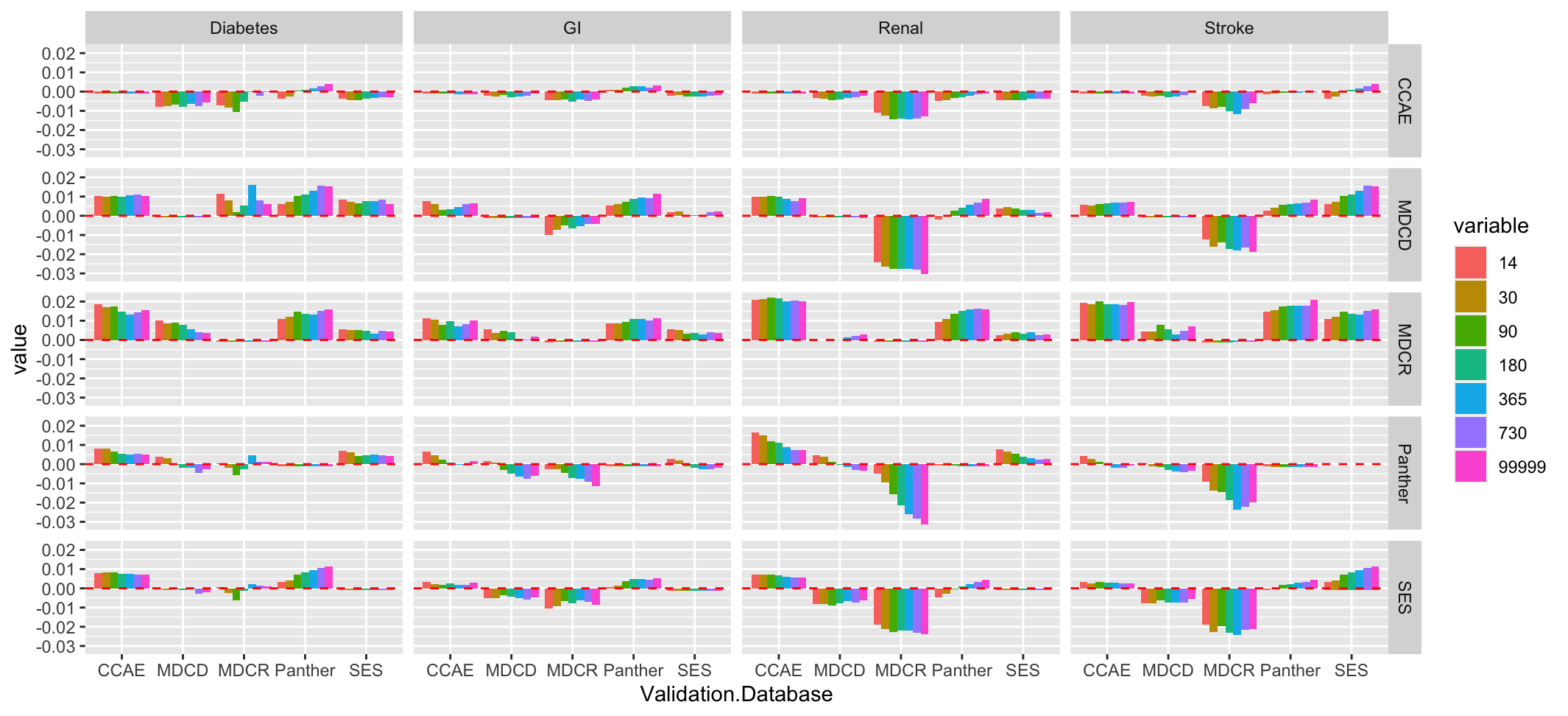


Figure 1- Calibration intercept values across models developed using the different lookbacks. The x-axis is the validation database, the y-axis is the calibration intercept, the grid rows are the development database and the grid columns are the outcomes. The red dashed line corresponds to 0, a perfect calibration intercept.

Calibration (slope) plot for internal and external validation


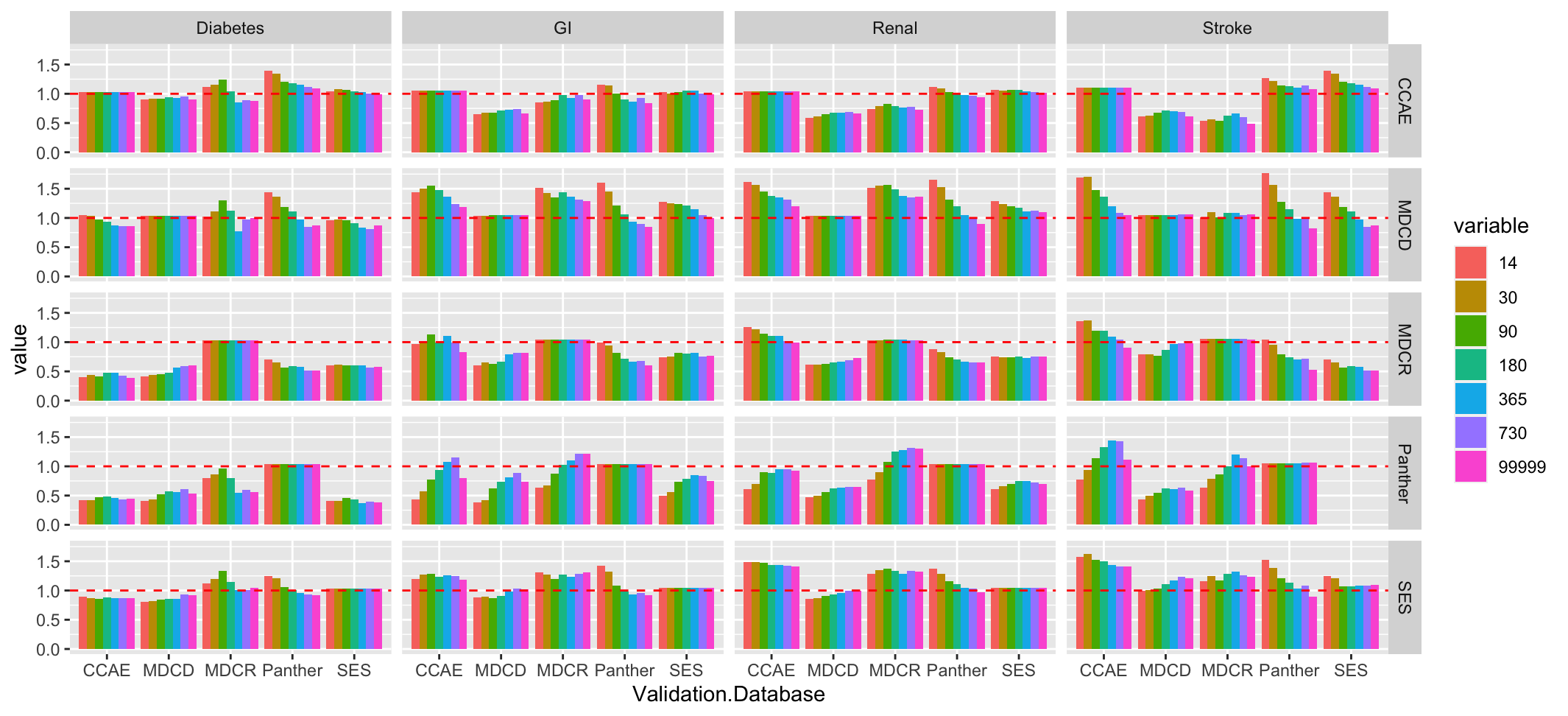


Figure 2 - Calibration slope values across models developed using the different lookbacks. The x-axis is the validation database, the y-axis is the calibration slope, the grid rows are the development database and the grid columns are the outcomes. The red dashed line corresponds to 1, a perfect calibration slope.

Calibration (intercept) for external validation

Table 1 – the calibration intercept for the models trained using covariates with different lookback periods externally validated across the other databases

|  |  |  | **Lookback (days)** | | | | | | |
| --- | --- | --- | --- | --- | --- | --- | --- | --- | --- |
| **Outcome** | **Validation Database** | **Development Database** | **14** | **30** | **90** | **180** | **365** | **730** | **All time** |
| Diabetes | CCAE | MDCD | 0.0102 | 0.0100 | 0.0104 | 0.0099 | 0.0105 | 0.0109 | 0.0105 |
| Diabetes | CCAE | MDCR | 0.0185 | 0.0170 | 0.0173 | 0.0147 | 0.0133 | 0.0143 | 0.0154 |
| Diabetes | CCAE | Panther | 0.0081 | 0.0080 | 0.0065 | 0.0053 | 0.0049 | 0.0054 | 0.0049 |
| Diabetes | CCAE | SES | 0.0080 | 0.0083 | 0.0082 | 0.0076 | 0.0074 | 0.0071 | 0.0070 |
| Diabetes | MDCD | CCAE | -0.0079 | -0.0075 | -0.0069 | -0.0078 | -0.0063 | -0.0076 | -0.0056 |
| Diabetes | MDCD | MDCR | 0.0100 | 0.0086 | 0.0089 | 0.0080 | 0.0056 | 0.0039 | 0.0038 |
| Diabetes | MDCD | Panther | 0.0038 | 0.0029 | -0.0002 | -0.0019 | -0.0020 | -0.0046 | -0.0026 |
| Diabetes | MDCD | SES | -0.0005 | -0.0010 | -0.0006 | -0.0009 | 0.0002 | -0.0029 | -0.0020 |
| Diabetes | MDCR | CCAE | -0.0070 | -0.0083 | -0.0107 | -0.0052 | -0.0003 | -0.0022 | -0.0004 |
| Diabetes | MDCR | MDCD | 0.0114 | 0.0080 | 0.0019 | 0.0053 | 0.0161 | 0.0080 | 0.0059 |
| Diabetes | MDCR | Panther | 0.0003 | -0.0020 | -0.0058 | -0.0025 | 0.0046 | 0.0014 | 0.0014 |
| Diabetes | MDCR | SES | 0.0008 | -0.0023 | -0.0063 | -0.0012 | 0.0023 | 0.0016 | 0.0010 |
| Diabetes | Panther | CCAE | -0.0038 | -0.0024 | 0.0006 | 0.0008 | 0.0017 | 0.0030 | 0.0039 |
| Diabetes | Panther | MDCD | 0.0061 | 0.0073 | 0.0104 | 0.0110 | 0.0129 | 0.0157 | 0.0154 |
| Diabetes | Panther | MDCR | 0.0110 | 0.0122 | 0.0146 | 0.0135 | 0.0134 | 0.0153 | 0.0158 |
| Diabetes | Panther | SES | 0.0035 | 0.0042 | 0.0073 | 0.0084 | 0.0096 | 0.0106 | 0.0114 |
| Diabetes | SES | CCAE | -0.0035 | -0.0046 | -0.0045 | -0.0038 | -0.0035 | -0.0029 | -0.0029 |
| Diabetes | SES | MDCD | 0.0082 | 0.0073 | 0.0064 | 0.0076 | 0.0076 | 0.0086 | 0.0063 |
| Diabetes | SES | MDCR | 0.0057 | 0.0050 | 0.0052 | 0.0050 | 0.0033 | 0.0050 | 0.0043 |
| Diabetes | SES | Panther | 0.0068 | 0.0063 | 0.0041 | 0.0046 | 0.0052 | 0.0045 | 0.0042 |
| GI | CCAE | MDCD | 0.0076 | 0.0059 | 0.0031 | 0.0033 | 0.0045 | 0.0059 | 0.0066 |
| GI | CCAE | MDCR | 0.0115 | 0.0106 | 0.0078 | 0.0098 | 0.0069 | 0.0082 | 0.0102 |
| GI | CCAE | Panther | 0.0064 | 0.0045 | 0.0022 | 0.0007 | -0.0002 | -0.0008 | 0.0016 |
| GI | CCAE | SES | 0.0035 | 0.0023 | 0.0019 | 0.0026 | 0.0019 | 0.0020 | 0.0028 |
| GI | MDCD | CCAE | -0.0021 | -0.0027 | -0.0018 | -0.0028 | -0.0027 | -0.0023 | -0.0010 |
| GI | MDCD | MDCR | 0.0057 | 0.0038 | 0.0047 | 0.0039 | 0.0006 | 0.0003 | 0.0016 |
| GI | MDCD | Panther | 0.0017 | 0.0007 | -0.0029 | -0.0051 | -0.0065 | -0.0075 | -0.0060 |
| GI | MDCD | SES | -0.0052 | -0.0049 | -0.0035 | -0.0043 | -0.0053 | -0.0058 | -0.0049 |
| GI | MDCR | CCAE | -0.0045 | -0.0044 | -0.0041 | -0.0054 | -0.0039 | -0.0047 | -0.0042 |
| GI | MDCR | MDCD | -0.0101 | -0.0074 | -0.0051 | -0.0065 | -0.0053 | -0.0044 | -0.0042 |
| GI | MDCR | Panther | -0.0025 | -0.0026 | -0.0047 | -0.0072 | -0.0074 | -0.0092 | -0.0114 |
| GI | MDCR | SES | -0.0106 | -0.0093 | -0.0065 | -0.0079 | -0.0063 | -0.0072 | -0.0085 |
| GI | Panther | CCAE | 0.0010 | 0.0008 | 0.0020 | 0.0028 | 0.0029 | 0.0022 | 0.0033 |
| GI | Panther | MDCD | 0.0052 | 0.0062 | 0.0073 | 0.0087 | 0.0096 | 0.0093 | 0.0113 |
| GI | Panther | MDCR | 0.0087 | 0.0087 | 0.0096 | 0.0108 | 0.0110 | 0.0100 | 0.0114 |
| GI | Panther | SES | 0.0005 | 0.0012 | 0.0037 | 0.0048 | 0.0049 | 0.0046 | 0.0051 |
| GI | SES | CCAE | -0.0022 | -0.0018 | -0.0024 | -0.0027 | -0.0026 | -0.0021 | -0.0019 |
| GI | SES | MDCD | 0.0017 | 0.0022 | 0.0004 | 0.0002 | 0.0003 | 0.0019 | 0.0024 |
| GI | SES | MDCR | 0.0057 | 0.0054 | 0.0032 | 0.0037 | 0.0030 | 0.0041 | 0.0037 |
| GI | SES | Panther | 0.0028 | 0.0019 | -0.0009 | -0.0018 | -0.0028 | -0.0028 | -0.0020 |
| Renal | CCAE | MDCD | 0.0100 | 0.0099 | 0.0102 | 0.0098 | 0.0087 | 0.0078 | 0.0091 |
| Renal | CCAE | MDCR | 0.0210 | 0.0214 | 0.0221 | 0.0215 | 0.0199 | 0.0205 | 0.0202 |
| Renal | CCAE | Panther | 0.0165 | 0.0151 | 0.0119 | 0.0113 | 0.0089 | 0.0075 | 0.0073 |
| Renal | CCAE | SES | 0.0073 | 0.0070 | 0.0071 | 0.0069 | 0.0060 | 0.0057 | 0.0056 |
| Renal | MDCD | CCAE | -0.0033 | -0.0038 | -0.0044 | -0.0041 | -0.0032 | -0.0030 | -0.0020 |
| Renal | MDCD | MDCR | -0.0001 | 0.0000 | -0.0002 | -0.0001 | 0.0015 | 0.0022 | 0.0030 |
| Renal | MDCD | Panther | 0.0047 | 0.0037 | 0.0013 | -0.0003 | -0.0015 | -0.0029 | -0.0033 |
| Renal | MDCD | SES | -0.0080 | -0.0083 | -0.0088 | -0.0077 | -0.0067 | -0.0073 | -0.0061 |
| Renal | MDCR | CCAE | -0.0110 | -0.0126 | -0.0144 | -0.0139 | -0.0144 | -0.0142 | -0.0130 |
| Renal | MDCR | MDCD | -0.0241 | -0.0263 | -0.0277 | -0.0276 | -0.0275 | -0.0280 | -0.0305 |
| Renal | MDCR | Panther | -0.0050 | -0.0095 | -0.0157 | -0.0216 | -0.0261 | -0.0282 | -0.0316 |
| Renal | MDCR | SES | -0.0187 | -0.0212 | -0.0226 | -0.0218 | -0.0220 | -0.0231 | -0.0238 |
| Renal | Panther | CCAE | -0.0050 | -0.0045 | -0.0035 | -0.0028 | -0.0021 | -0.0015 | -0.0009 |
| Renal | Panther | MDCD | -0.0019 | -0.0001 | 0.0026 | 0.0041 | 0.0059 | 0.0070 | 0.0088 |
| Renal | Panther | MDCR | 0.0095 | 0.0110 | 0.0138 | 0.0152 | 0.0160 | 0.0164 | 0.0160 |
| Renal | Panther | SES | -0.0046 | -0.0029 | -0.0005 | 0.0011 | 0.0023 | 0.0032 | 0.0046 |
| Renal | SES | CCAE | -0.0046 | -0.0044 | -0.0046 | -0.0046 | -0.0039 | -0.0038 | -0.0037 |
| Renal | SES | MDCD | 0.0040 | 0.0045 | 0.0038 | 0.0030 | 0.0031 | 0.0015 | 0.0018 |
| Renal | SES | MDCR | 0.0025 | 0.0032 | 0.0040 | 0.0034 | 0.0041 | 0.0027 | 0.0028 |
| Renal | SES | Panther | 0.0078 | 0.0066 | 0.0054 | 0.0039 | 0.0029 | 0.0023 | 0.0026 |
| Stroke | CCAE | MDCD | 0.0059 | 0.0052 | 0.0061 | 0.0064 | 0.0067 | 0.0071 | 0.0072 |
| Stroke | CCAE | MDCR | 0.0194 | 0.0188 | 0.0202 | 0.0187 | 0.0184 | 0.0183 | 0.0199 |
| Stroke | CCAE | Panther | 0.0042 | 0.0026 | 0.0011 | -0.0006 | -0.0017 | -0.0019 | -0.0009 |
| Stroke | CCAE | SES | 0.0031 | 0.0026 | 0.0032 | 0.0030 | 0.0028 | 0.0027 | 0.0027 |
| Stroke | MDCD | CCAE | -0.0022 | -0.0024 | -0.0023 | -0.0029 | -0.0024 | -0.0017 | -0.0001 |
| Stroke | MDCD | MDCR | 0.0043 | 0.0045 | 0.0079 | 0.0056 | 0.0031 | 0.0049 | 0.0072 |
| Stroke | MDCD | Panther | 0.0002 | -0.0010 | -0.0017 | -0.0030 | -0.0040 | -0.0043 | -0.0035 |
| Stroke | MDCD | SES | -0.0077 | -0.0076 | -0.0061 | -0.0073 | -0.0075 | -0.0074 | -0.0054 |
| Stroke | MDCR | CCAE | -0.0077 | -0.0086 | -0.0078 | -0.0103 | -0.0116 | -0.0092 | -0.0061 |
| Stroke | MDCR | MDCD | -0.0123 | -0.0163 | -0.0138 | -0.0175 | -0.0181 | -0.0165 | -0.0187 |
| Stroke | MDCR | Panther | -0.0091 | -0.0138 | -0.0144 | -0.0188 | -0.0238 | -0.0223 | -0.0200 |
| Stroke | MDCR | SES | -0.0190 | -0.0226 | -0.0196 | -0.0233 | -0.0241 | -0.0214 | -0.0212 |
| Stroke | Panther | CCAE | -0.0015 | -0.0012 | -0.0007 | -0.0006 | -0.0005 | -0.0004 | -0.0001 |
| Stroke | Panther | MDCD | 0.0028 | 0.0040 | 0.0057 | 0.0060 | 0.0067 | 0.0069 | 0.0084 |
| Stroke | Panther | MDCR | 0.0146 | 0.0155 | 0.0175 | 0.0180 | 0.0178 | 0.0179 | 0.0210 |
| Stroke | Panther | SES | -0.0010 | 0.0001 | 0.0016 | 0.0022 | 0.0031 | 0.0032 | 0.0045 |
| Stroke | SES | CCAE | -0.0038 | -0.0024 | 0.0006 | 0.0008 | 0.0017 | 0.0030 | 0.0039 |
| Stroke | SES | MDCD | 0.0061 | 0.0073 | 0.0104 | 0.0110 | 0.0129 | 0.0157 | 0.0154 |
| Stroke | SES | MDCR | 0.0110 | 0.0122 | 0.0146 | 0.0135 | 0.0134 | 0.0153 | 0.0158 |
| Stroke | SES | SES | 0.0035 | 0.0042 | 0.0073 | 0.0084 | 0.0096 | 0.0106 | 0.0114 |

Calibration (slope) for external validation

Table 2 -the calibration slope for the models trained using covariates with different lookback periods externally validated across the other databases

|  |  |  | **Lookback (days)** | | | | | | |
| --- | --- | --- | --- | --- | --- | --- | --- | --- | --- |
| **Outcome** | **Validation Database** | **Development Database** | **14** | **30** | **90** | **180** | **365** | **730** | **All time** |
| Diabetes | CCAE | MDCD | 1.0457 | 1.0311 | 0.9731 | 0.9315 | 0.8749 | 0.8545 | 0.8596 |
| Diabetes | CCAE | MDCR | 0.3968 | 0.4401 | 0.4102 | 0.4792 | 0.4779 | 0.4258 | 0.3838 |
| Diabetes | CCAE | Panther | 0.4262 | 0.4275 | 0.4655 | 0.4834 | 0.4575 | 0.4314 | 0.4463 |
| Diabetes | CCAE | SES | 0.8884 | 0.8746 | 0.8610 | 0.8803 | 0.8724 | 0.8679 | 0.8756 |
| Diabetes | MDCD | CCAE | 0.9073 | 0.9097 | 0.9152 | 0.9469 | 0.9325 | 0.9522 | 0.9076 |
| Diabetes | MDCD | MDCR | 0.4130 | 0.4452 | 0.4531 | 0.4821 | 0.5634 | 0.5941 | 0.6044 |
| Diabetes | MDCD | Panther | 0.4026 | 0.4329 | 0.5266 | 0.5750 | 0.5608 | 0.6054 | 0.5348 |
| Diabetes | MDCD | SES | 0.8018 | 0.8231 | 0.8389 | 0.8589 | 0.8618 | 0.9313 | 0.9181 |
| Diabetes | MDCR | CCAE | 1.1232 | 1.1606 | 1.2455 | 1.0412 | 0.8527 | 0.8950 | 0.8761 |
| Diabetes | MDCR | MDCD | 1.0220 | 1.1077 | 1.2958 | 1.1210 | 0.7692 | 0.9792 | 1.0047 |
| Diabetes | MDCR | Panther | 0.7931 | 0.8595 | 0.9616 | 0.8038 | 0.5425 | 0.5917 | 0.5658 |
| Diabetes | MDCR | SES | 1.1209 | 1.1996 | 1.3343 | 1.1473 | 1.0047 | 1.0081 | 1.0456 |
| Diabetes | Panther | CCAE | 1.3999 | 1.3386 | 1.2006 | 1.1863 | 1.1550 | 1.1221 | 1.0945 |
| Diabetes | Panther | MDCD | 1.4416 | 1.3685 | 1.1845 | 1.1151 | 0.9705 | 0.8490 | 0.8766 |
| Diabetes | Panther | MDCR | 0.7047 | 0.6568 | 0.5632 | 0.5945 | 0.5822 | 0.5172 | 0.5087 |
| Diabetes | Panther | SES | 1.2476 | 1.2039 | 1.0614 | 1.0080 | 0.9519 | 0.9310 | 0.9167 |
| Diabetes | SES | CCAE | 1.0445 | 1.0732 | 1.0630 | 1.0406 | 1.0252 | 1.0025 | 0.9961 |
| Diabetes | SES | MDCD | 0.9647 | 0.9696 | 0.9552 | 0.9118 | 0.8378 | 0.8102 | 0.8706 |
| Diabetes | SES | MDCR | 0.6053 | 0.6181 | 0.6015 | 0.6012 | 0.6050 | 0.5611 | 0.5765 |
| Diabetes | SES | Panther | 0.4057 | 0.4113 | 0.4582 | 0.4324 | 0.3712 | 0.3953 | 0.3838 |
| GI | CCAE | MDCD | 1.4381 | 1.5003 | 1.5571 | 1.4753 | 1.3623 | 1.2330 | 1.1817 |
| GI | CCAE | MDCR | 0.9741 | 1.0055 | 1.1274 | 0.9929 | 1.1022 | 0.9885 | 0.8327 |
| GI | CCAE | Panther | 0.4395 | 0.5760 | 0.7720 | 0.9392 | 1.0721 | 1.1504 | 0.7975 |
| GI | CCAE | SES | 1.2017 | 1.2713 | 1.2850 | 1.2390 | 1.2603 | 1.2462 | 1.1808 |
| GI | MDCD | CCAE | 0.6458 | 0.6801 | 0.6711 | 0.7152 | 0.7320 | 0.7362 | 0.6685 |
| GI | MDCD | MDCR | 0.6023 | 0.6546 | 0.6322 | 0.6642 | 0.7911 | 0.8180 | 0.8140 |
| GI | MDCD | Panther | 0.3810 | 0.4247 | 0.6205 | 0.7377 | 0.8064 | 0.8884 | 0.7352 |
| GI | MDCD | SES | 0.8823 | 0.8892 | 0.8688 | 0.9090 | 0.9804 | 1.0312 | 1.0164 |
| GI | MDCR | CCAE | 0.8497 | 0.8604 | 0.8934 | 0.9731 | 0.9335 | 0.9780 | 0.9066 |
| GI | MDCR | MDCD | 1.5144 | 1.4274 | 1.3508 | 1.4414 | 1.3633 | 1.3160 | 1.2907 |
| GI | MDCR | Panther | 0.6333 | 0.6722 | 0.8733 | 1.0253 | 1.1047 | 1.2176 | 1.2148 |
| GI | MDCR | SES | 1.3104 | 1.2685 | 1.1911 | 1.2721 | 1.2351 | 1.2848 | 1.3154 |
| GI | Panther | CCAE | 1.1609 | 1.1422 | 1.0057 | 0.9070 | 0.8696 | 0.9274 | 0.8447 |
| GI | Panther | MDCD | 1.6044 | 1.4524 | 1.2176 | 1.0586 | 0.9350 | 0.8984 | 0.8503 |
| GI | Panther | MDCR | 0.9949 | 0.9412 | 0.8143 | 0.7168 | 0.6631 | 0.6836 | 0.6022 |
| GI | Panther | SES | 1.4290 | 1.3261 | 1.0868 | 0.9828 | 0.9320 | 0.9578 | 0.9180 |
| GI | SES | CCAE | 1.0329 | 1.0091 | 1.0267 | 1.0521 | 1.0515 | 1.0012 | 0.9888 |
| GI | SES | MDCD | 1.2787 | 1.2541 | 1.2361 | 1.2150 | 1.1510 | 1.0442 | 0.9974 |
| GI | SES | MDCR | 0.7460 | 0.7550 | 0.8173 | 0.8026 | 0.8169 | 0.7552 | 0.7672 |
| GI | SES | Panther | 0.4979 | 0.5616 | 0.7334 | 0.7916 | 0.8493 | 0.8355 | 0.7463 |
| Renal | CCAE | MDCD | 1.6098 | 1.5633 | 1.4556 | 1.3717 | 1.3457 | 1.3110 | 1.2020 |
| Renal | CCAE | MDCR | 1.2527 | 1.2202 | 1.1445 | 1.1114 | 1.1031 | 1.0177 | 0.9907 |
| Renal | CCAE | Panther | 0.6070 | 0.7005 | 0.9041 | 0.8818 | 0.9533 | 0.9525 | 0.9269 |
| Renal | CCAE | SES | 1.4871 | 1.4839 | 1.4687 | 1.4379 | 1.4379 | 1.4254 | 1.4100 |
| Renal | MDCD | CCAE | 0.5921 | 0.6098 | 0.6491 | 0.6752 | 0.6816 | 0.6834 | 0.6652 |
| Renal | MDCD | MDCR | 0.6108 | 0.6140 | 0.6331 | 0.6553 | 0.6671 | 0.6880 | 0.7231 |
| Renal | MDCD | Panther | 0.4721 | 0.4967 | 0.5627 | 0.6243 | 0.6341 | 0.6418 | 0.6427 |
| Renal | MDCD | SES | 0.8591 | 0.8757 | 0.9069 | 0.9279 | 0.9576 | 0.9907 | 1.0002 |
| Renal | MDCR | CCAE | 0.7437 | 0.7848 | 0.8255 | 0.7954 | 0.7631 | 0.7761 | 0.7319 |
| Renal | MDCR | MDCD | 1.5157 | 1.5560 | 1.5627 | 1.4892 | 1.3706 | 1.3484 | 1.3628 |
| Renal | MDCR | Panther | 0.7772 | 0.9013 | 1.0790 | 1.2542 | 1.2769 | 1.3114 | 1.3064 |
| Renal | MDCR | SES | 1.2872 | 1.3501 | 1.3725 | 1.3412 | 1.2898 | 1.3373 | 1.3216 |
| Renal | Panther | CCAE | 1.1228 | 1.0935 | 1.0281 | 1.0029 | 0.9764 | 0.9713 | 0.9412 |
| Renal | Panther | MDCD | 1.6549 | 1.5244 | 1.3131 | 1.2026 | 1.0480 | 0.9828 | 0.9029 |
| Renal | Panther | MDCR | 0.8781 | 0.8247 | 0.7371 | 0.6987 | 0.6598 | 0.6581 | 0.6589 |
| Renal | Panther | SES | 1.3688 | 1.2901 | 1.1653 | 1.1053 | 1.0400 | 1.0322 | 0.9702 |
| Renal | SES | CCAE | 1.0630 | 1.0501 | 1.0660 | 1.0722 | 1.0412 | 1.0324 | 1.0198 |
| Renal | SES | MDCD | 1.2938 | 1.2400 | 1.2019 | 1.1733 | 1.1164 | 1.1190 | 1.0997 |
| Renal | SES | MDCR | 0.7583 | 0.7455 | 0.7393 | 0.7521 | 0.7341 | 0.7556 | 0.7528 |
| Renal | SES | Panther | 0.6052 | 0.6551 | 0.7017 | 0.7469 | 0.7506 | 0.7215 | 0.6954 |
| Stroke | CCAE | MDCD | 1.6879 | 1.7052 | 1.4803 | 1.3611 | 1.1948 | 1.0917 | 1.0531 |
| Stroke | CCAE | MDCR | 1.3564 | 1.3691 | 1.1890 | 1.2004 | 1.0891 | 1.0452 | 0.9106 |
| Stroke | CCAE | Panther | 0.7721 | 0.9390 | 1.1415 | 1.3339 | 1.4420 | 1.4351 | 1.1180 |
| Stroke | CCAE | SES | 1.5810 | 1.6234 | 1.5207 | 1.4968 | 1.4300 | 1.4079 | 1.4097 |
| Stroke | MDCD | CCAE | 0.6074 | 0.6218 | 0.6711 | 0.7112 | 0.7074 | 0.6846 | 0.6113 |
| Stroke | MDCD | MDCR | 0.7905 | 0.7893 | 0.7667 | 0.8629 | 0.9739 | 0.9812 | 0.9774 |
| Stroke | MDCD | Panther | 0.4340 | 0.4962 | 0.5515 | 0.6224 | 0.6162 | 0.6392 | 0.5876 |
| Stroke | MDCD | SES | 1.0003 | 1.0124 | 1.0327 | 1.1130 | 1.1778 | 1.2319 | 1.2118 |
| Stroke | MDCR | CCAE | 0.5427 | 0.5600 | 0.5399 | 0.6211 | 0.6685 | 0.6063 | 0.4903 |
| Stroke | MDCR | MDCD | 1.0138 | 1.1007 | 1.0159 | 1.0855 | 1.0907 | 1.0445 | 1.0594 |
| Stroke | MDCR | Panther | 0.6357 | 0.7831 | 0.8612 | 1.0055 | 1.2039 | 1.1388 | 0.9976 |
| Stroke | MDCR | SES | 1.1557 | 1.2474 | 1.1682 | 1.2845 | 1.3167 | 1.2547 | 1.2367 |
| Stroke | Panther | CCAE | 1.2718 | 1.2224 | 1.1453 | 1.1282 | 1.1056 | 1.1416 | 1.0754 |
| Stroke | Panther | MDCD | 1.7630 | 1.5648 | 1.2736 | 1.1490 | 0.9914 | 1.0014 | 0.8222 |
| Stroke | Panther | MDCR | 1.0391 | 0.9510 | 0.7955 | 0.7416 | 0.6978 | 0.7209 | 0.5271 |
| Stroke | Panther | SES | 1.5227 | 1.3868 | 1.2057 | 1.1354 | 1.0300 | 1.0860 | 0.8997 |
| Stroke | SES | CCAE | 1.3999 | 1.3386 | 1.2006 | 1.1863 | 1.1550 | 1.1221 | 1.0945 |
| Stroke | SES | MDCD | 1.4416 | 1.3685 | 1.1845 | 1.1151 | 0.9705 | 0.8490 | 0.8766 |
| Stroke | SES | MDCR | 0.7047 | 0.6568 | 0.5632 | 0.5945 | 0.5822 | 0.5172 | 0.5087 |
| Stroke | SES | SES | 1.2476 | 1.2039 | 1.0614 | 1.0080 | 0.9519 | 0.9310 | 0.9167 |
