# Supplementary material for: Evaluating the impact of covariate lookback times on performance of patient-level prediction models
Source: BMC Med Res Methodol. 2021 Aug 28;21:180. doi: 10.1186/s12874-021-01370-2 (PMC8403343; doi:10.1186/s12874-021-01370-2)
Supplement: Supplementary file 5 — Additional file 5. [file 12874_2021_1370_MOESM5_ESM.docx]

**Figure 4. Mean number of predictors in internally validated models by lookback across two chronic (Diabetes and Renal Impairment) and two acute (Gastrointestinal bleeding and Stroke) cohorts over five US databases.**


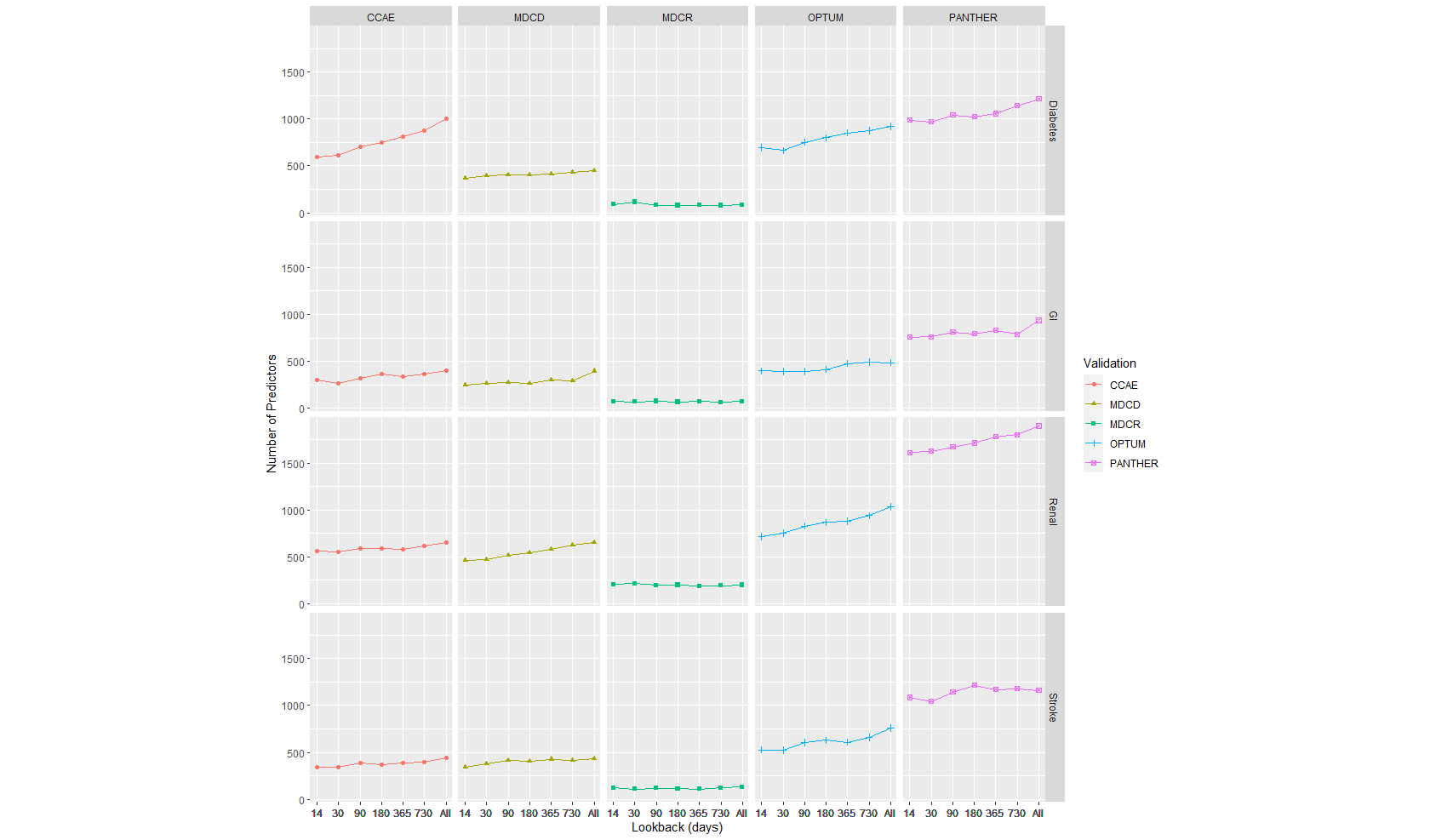


Database
