# Supplementary material for: Evaluating the impact of covariate lookback times on performance of patient-level prediction models
Source: BMC Med Res Methodol. 2021 Aug 28;21:180. doi: 10.1186/s12874-021-01370-2 (PMC8403343; doi:10.1186/s12874-021-01370-2)

**Figure 5. Mean AUPRC across two chronic (Diabetes and Renal Impairment) and two acute (Gastrointestinal bleeding and Stroke) cohorts over five US databases. The database at the top was used to train the model. Colors and shapes indicate the database used to compute the AUPRC.**


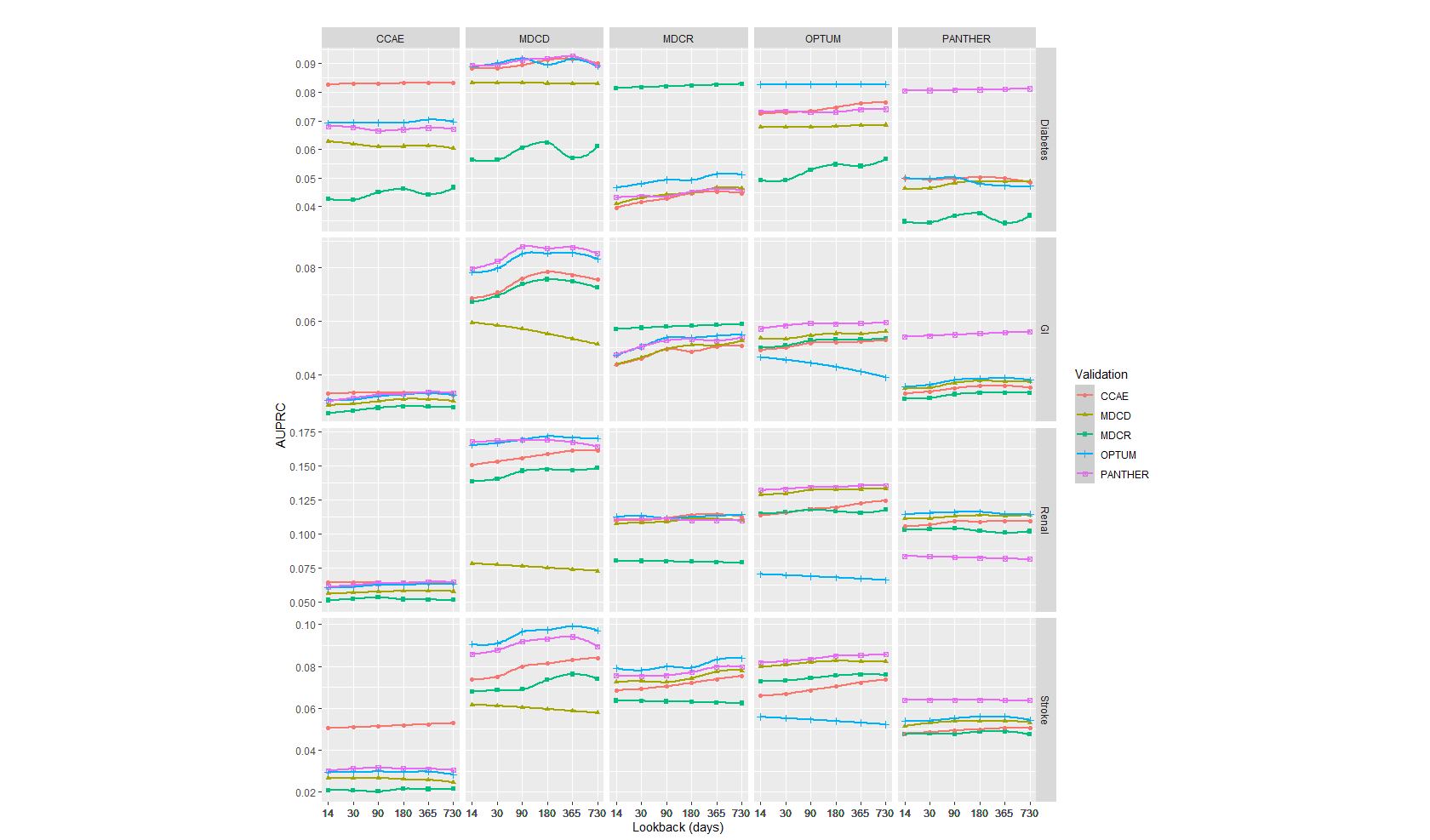

Supplement: Supplementary file 6 — Additional file 6. [file 12874_2021_1370_MOESM6_ESM.docx]
